# The PML-Interacting Protein DAXX: Histone Loading Gets into the Picture

**DOI:** 10.3389/fonc.2013.00152

**Published:** 2013-06-07

**Authors:** Paolo Salomoni

**Affiliations:** ^1^Samantha Dickson Brain Cancer Unit, UCL Cancer Institute, University College London, London, UK

**Keywords:** PML, DAXX, cancer, epigenetics, histone variant

## Abstract

The promyelocytic leukemia (PML) protein has been implicated in regulation of multiple key cellular functions, from transcription to calcium homeostasis. PML pleiotropic role is in part related to its ability to localize to both the nucleus and cytoplasm. In the nucleus, PML is known to regulate gene transcription, a role linked to its ability to associate with transcription factors as well as chromatin-remodelers. A new twist came from the discovery that the PML-interacting protein death-associated protein 6 (DAXX) acts as chaperone for the histone H3.3 variant. H3.3 is found enriched at active genes, centromeric heterochromatin, and telomeres, and has been proposed to act as important carrier of epigenetic information. Our recent work has implicated DAXX in regulation of H3.3 loading and transcription in the central nervous system (CNS). Remarkably, driver mutations in H3.3 and/or its loading machinery have been identified in brain cancer, thus suggesting a role for altered H3.3 function/deposition in CNS tumorigenesis. Aberrant H3.3 deposition may also play a role in leukemia pathogenesis, given DAXX role in PML-RARα-driven transformation and the identification of a DAXX missense mutation in acute myeloid leukemia. This review aims to critically discuss the existing literature and propose new avenues for investigation.

## The Promyelocytic Leukemia Protein

The Promyelocytic Leukemia (*PML*) gene was originally identified at the breakpoint of the t(15;17) translocation of Acute Promyelocytic Leukemia (APL), which generates the PML/retinoic acid receptor (RAR)α oncogene, an inhibitor of PML and RARα functions (Salomoni et al., 2008) [please refer to accompanying articles^1^ and reviews in the field, e.g. (Grimwade and Solomon, 1997; Brown et al., 2009; de The and Chen, 2010), for detailed information on APL pathogenesis]. PML can localize to the cytoplasm [for more extensive discussion on the role of cytoplasmic PML, see (Lin et al., 2004; Giorgi et al., 2010; Pinton et al., 2011) as well as this issue of Frontiers] and the nucleus, where it forms the PML nuclear body (PML-NB), of which it is the essential component (Salomoni and Khelifi, 2006; Salomoni et al., 2008). The PML-NB is a subnuclear structure associated with storage and post-translational modifications (PTMs) of several nuclear factors [(Salomoni et al., 2008) for more extensive discussion on the role and regulation of PML-NBs, see comprehensive reviews in the field (Zhong et al., 2000b; Bernardi and Pandolfi, 2007; Lallemand-Breitenbach and de The, 2012), including this issue of Frontiers]. The PML-NB is disrupted in APL cells by PML/RARα (Salomoni et al., 2008). Both PML and PML/RARα (via the PML moiety) can be targeted pharmacologically using arsenic trioxide (ATO), which, in part through direct binding, promotes their ubiquitin-dependent degradation (Jeanne et al., 2010; Zhang et al., 2010). ATO is used in APL therapy because of its ability to target the leukemic stem cell pool (de The and Chen, 2010).

Although the PML gene is rarely mutated in cancer, its protein expression is lost in a number of human tumors, suggesting that it acts as tumor suppressor. Indeed, PML limits tumorigenesis in APL, lung, and prostate cancer models (Salomoni and Pandolfi, 2002). However, recent studies have highlighted a potential role of PML in established tumors. In this respect, PML is required for maintenance of the leukemia-initiating stem cell pool in chronic myeloid leukemia (Ito et al., 2008). Notably, ATO phenocopies the effect of PML loss in leukemic stem cells and requires PML for this effect (Ito et al., 2008). Another growth suppressor, p21 controls leukemic stem cell maintenance via regulation of genomic stability (Viale et al., 2009). Furthermore, an additional study from the Pandolfi’s group showed that PML plays an important pro-survival role in cancer via regulation of tumor cell metabolism [(Carracedo et al., 2012); for more extensive discussion on the role of PML in tumorigenesis, see comprehensive reviews in the field (Salomoni and Pandolfi, 2002), including this issue of Frontiers].

What is PML nuclear function(s)? Several studies have implicated PML in regulation of transcription (Zhong et al., 2000b; Bernardi and Pandolfi, 2007; Salomoni et al., 2008). In this respect, PML-NBs localize in the proximity of active transcription sites in a cell cycle-dependent manner (Kiesslich et al., 2002). Notably, PML can directly regulate the function of several transcription factors (Bernardi and Pandolfi, 2007). For instance, PML interaction with the p53 tumor suppressor promotes p53-dependent transcription in a PML-NB-dependent as well as – independent manner (Bischof et al., 2002; Bernardi et al., 2004; Bernardi and Pandolfi, 2007; Salomoni et al., 2008). Furthermore, work from our group and others have shown that the tumor suppressor and transcriptional repressor retinoblastoma (pRb) also localizes to PML-NBs (Alcalay et al., 1998), resulting in alterations of its phosphorylation status (Ferbeyre et al., 2000; Regad et al., 2009). Interestingly, not only transcription factors are found in PML-NBs, as a number of chromatin regulators localize to these structures, such as the histone acetyltransferase CREB-binding protein (CBP)/p300, which can acetylate histones as well as transcription factors. In this respect, it has been proposed that in senescent cells PML promotes p53 acetylation via dynamic localization of CBP to PML-NBs (Pearson et al., 2000). It is presently unknown whether PML could affect CBP-mediated acetylation of histone tails, in addition to transcription factors. It is important to note that PML-NBs contain chromatin-associated factors with repressive activity, such as histone deacetylase 1 (HDAC1), the corepressors N-Cor, Sin3A (Khan et al., 2001), and the heterochromatin-associated protein 1 (HP1) (Seeler et al., 1998). Together, these findings suggest that PML could serve as scaffold for multiple chromatin-remodeling complexes, with potential implications for both transcriptional activation and repression. Interestingly, there is evidence that PML-NBs might be involved in regulation of chromatin architecture, as some genetic loci are non-randomly associated with the periphery of PML-NBs (Torok et al., 2009). Furthermore, PML has been implicated in special AT-rich sequence-binding protein 1 (SATB1)-mediated regulation of chromatin architecture and gene expression (Kumar et al., 2007). Although these studies suggest an involvement of PML in chromatin regulation via interaction with histone-modifying enzymes and other chromatin regulators, our understanding of PML and PML-NB role in this context remains limited.

New exciting studies now link PML to the histone loading machinery, with implications for chromatin remodeling and cancer pathogenesis. This will be the main focus of the present review article.

## The PML-Interacting Protein DAXX is a Chaperone for the Histone Variant H3.3

The death-associated protein 6 (DAXX) interacts with PML and is found in PML-NBs as well as heterochromatin (Khelifi et al., 2005; Salomoni and Khelifi, 2006). DAXX recruitment to PML-NBs occurs via binding to SUMOylated PML with DAXX SUMO-interacting motif (SIM) (Zhong et al., 2000a; Lin et al., 2006). DAXX SIM is also required for its ability to localize to heterochromatin [(Kuo et al., 2005) and our unpublished data]. DAXX loss results in embryonic lethality (Michaelson et al., 1999; Garrick et al., 2006), indicating an essential role in embryogenesis. DAXX was originally identified as a CD95-interacting protein in the cytoplasm, affecting CD95-dependent activation of c-Jun-N-terminal kinase (JNK) (Yang et al., 1997). The link with JNK was reported by subsequent studies, which implicated an apoptosis signal-regulating kinase 1 (ASK1)-dependent mechanism for DAXX-mediated JNK activation (Ko et al., 2001; Perlman et al., 2001; Khelifi et al., 2005). However, it is presently unclear whether endogenous DAXX localizes to the cytoplasm in physiological conditions [for discussion see (Lindsay et al., 2009)].

What would be DAXX nuclear function? DAXX has been shown to regulate transcription, either indirectly or directly. On one hand, DAXX destabilizes p53 via inhibition of mouse double minute 2 homolog (MDM2) ubiquitylation, thus resulting in repression of p53 target gene expression (Tang et al., 2006). On the other hand, DAXX regulates transcription of mammalian and viral genes via its ability to interact with a number of transcription factors [for more details see (Preston and Nicholl, 2006; Saffert and Kalejta, 2006; Salomoni and Khelifi, 2006; Lindsay et al., 2008; Lukashchuk and Everett, 2010; Tsai et al., 2011; Rivera-Molina et al., 2012; Glass and Everett, 2013; Schreiner et al., 2013; Shalginskikh et al., 2013)] and epigenetic regulators. In particular, DAXX interacts with HDAC-II (Hollenbach et al., 2002), acetyltransferases (CBP) (Kuo et al., 2005), and DNA methyltransferase (Dnmt1) (Puto and Reed, 2008; Zhang et al., 2013), suggesting an important role in chromatin remodeling.

**Figure 1:**
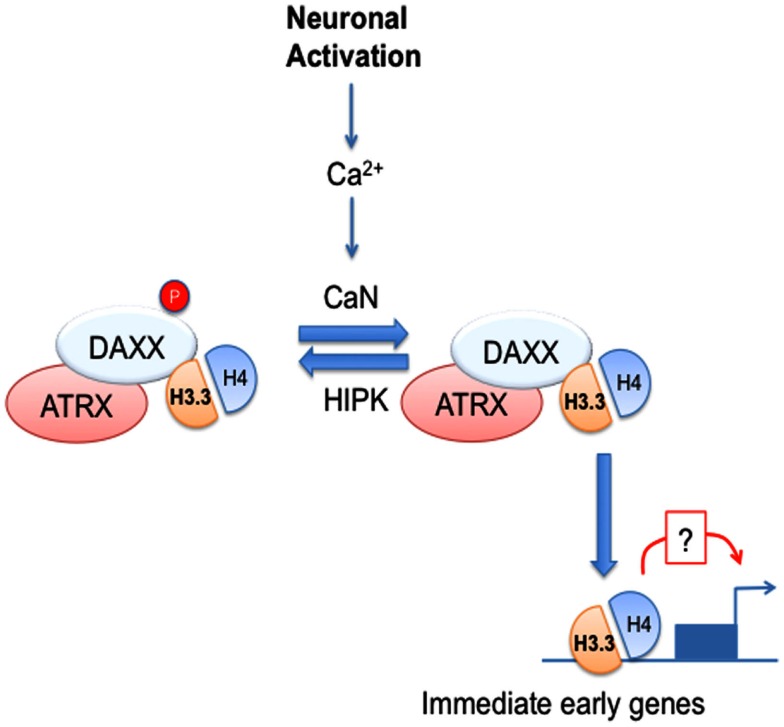
**DAXX chaperone activity is regulated by calcium-dependent signaling in neural cells**. Neuronal activation leads to calcium (Ca^2+^) entry and activation of the Ca^2+^-dependent phosphatase, calcineurin (CaN). In turn, CaN dephosphorylates DAXX at serine 669, leading to increased H3.3 loading at selected immediate early genes (IEGs). DAXX loss not only affects H3.3 loading, but also leads to impaired induction of IEGs, thus suggesting that H3.3 loading may modulate IEG transcriptional induction.

Recent exciting studies have implicated DAXX in direct chromatin regulation via its ability to act as chaperone for a histone 3 (H3) variant called H3.3. Best understood for PTMs of histones, chromatin modification also occurs via incorporation of histone variants. Unlike canonical H3, H3.3 can be loaded on DNA in a replication-independent manner. H3.3 is believed to be an important carrier of epigenetic information (Szenker et al., 2011). H3.3 is encoded by two genes, *H3F3A* and *H3F3B*. *H3F3A* inactivation via gene trap leads to perinatal lethality (Couldrey et al., 1999), whereas *H3F3B* knockout embryos display partial embryonic lethality and infertility in surviving homozygous animals (Bush et al., 2013). DAXX acts as a H3.3 chaperone as part of a nuclear complex containing the α-thalassemia and mental retardation X-linked (ATRX) DNA helicase (Drané et al., 2010; Lewis et al., 2010; Dawson and Kouzarides, 2012). ATRX, like DAXX, can associate with PML-NBs (Bérubé et al., 2007) and has been proposed to contribute to DAXX/H3.3 targeting to chromatin, potentially via its ability to bind histone repressive marks in heterochromatin and G-rich DNA repeats (Elsaesser et al., 2010; Law et al., 2010; Iwase et al., 2011). DAXX and ATRX mediate H3.3 loading onto telomeres and pericentric heterochromatin, with implications for transcription of telomeric and centromeric repeats (Drané et al., 2010; Goldberg et al., 2010; Lewis et al., 2010). Furthermore, H3.3 loading at telomeres has been suggested to play an important role in maintaining chromatin structure (Wong et al., 2009, 2010). Loading of H3.3 may affect transcription also at euchromatin, as it is enriched at transcriptionally active genes and has been proposed to regulate epigenetic memory of transcriptional competence (Henikoff, 2008; Ng and Gurdon, 2008; Jullien et al., 2012). Loading of H3.3 at transcription start site (TSS) and body of active gene is dependent on the chaperone HIRA (Goldberg et al., 2010). However, H3.3 is also enriched at regulatory regions not immediately adjacent to TSS (Mito et al., 2007; Jin et al., 2009; Goldberg et al., 2010). Deposition at those sites is in part HIRA-independent (Goldberg et al., 2010), but the histone chaperone involved was not known. In this respect, our recent work implicated DAXX in the regulation of H3.3 deposition at promoters and enhancers of immediate early genes (IEGs) in neurons (Michod et al., 2012), thus demonstrating that DAXX is one of the previously unidentified H3.3 chaperones at regulatory regions (Michod et al., 2012). Work from Genevieve Almouzni, John Gurdon, and Peter Adams groups (Ray-Gallet et al., 2011; Jullien et al., 2012; Pchelintsev et al., 2013) showed that HIRA could also mediate H3.3 loading at regulatory regions. Notably, DAXX-dependent H3.3 deposition correlates with its ability to modulate transcription, thus suggesting a link between H3.3 loading and transcription (Michod et al., 2012). Among the IEGs analyzed, only a subset of them displayed dependence on DAXX for H3.3 loading and transcriptional activation, thus suggesting that other H3.3 chaperones are involved in IEG regulation in neurons, such HIRA or DEK (Sawatsubashi et al., 2010; Jullien et al., 2012). Finally, both DAXX-dependent loading and transcription are controlled by a calcium-dependent phosphorylation switch affecting serine 669 (S669) (Michod et al., 2012), which is a target of homeodomain-interacting protein kinases (HIPKs) (Hofmann et al., 2003) (Figure 1). In particular, upon neuronal activation DAXX S669 is dephosphorylated by the calcium-dependent phosphatase calcineurin (CaN), leading to increased loading activity and transcription (Michod et al., 2012). Although H3.3 is preferentially found associated with hypophosphorylated DAXX, S669 dephosphorylation does not affect DAXX affinity for H3.3, suggesting that when in complex with H3.3 DAXX is either more effectively dephosphorylated or its HIPK-dependent phosphorylation is inhibited. Since CaN is believed to be mainly cytosolic, it is most likely that DAXX dephosphorylation occurs outside the nucleus, whereas one could speculate that its HIPK-dependent phosphorylation could be nuclear. It is important to note that DAXX S669 phosphorylation status does not affect its chromatin association. One could speculate that HIPKs could associate with DAXX on chromatin and inhibit its chaperone activity. Interestingly, the HIRA chaperone complex contains the CaN-binding protein CABIN1, a CaN regulator (Rai et al., 2011), suggesting that calcium-dependent signaling could regulate multiple H3.3 chaperone complexes.

Together, these studies suggest that DAXX-mediated loading of H3.3 at regulatory regions may affect transcription. One could argue that DAXX ability to regulate transcription could be H3.3-independent, for instance via its interaction with HDAC-II (Hollenbach et al., 2002), CBP (Kuo et al., 2005), or Dnmt1 (Puto and Reed, 2008; Zhang et al., 2013). However, DAXX loss fails to promote any significant changes in histone acetylation or DNA methylation at the BDNF Exon IV promoter (Michod et al., 2012). Another possibility is that DAXX regulates key transcription factors involved in activity-dependent IEG induction, in particular CREB and MEF2 (Hong et al., 2005; Flavell et al., 2008). For instance, DAXX has been recently reported to repress CREB transcriptional activity via direct interaction via its C-terminus (Huang et al., 2012) and HIPK2 is known to phosphorylate CREB (Sakamoto et al., 2010). However, based on these findings DAXX loss would result in increased CREB-mediated transcription, opposite to what we have observed in neurons (Michod et al., 2012). To incontrovertibly assess the role of DAXX-mediated H3.3 loading in transcription, one should test the ability of recently described DAXX mutants impaired in histone binding (Eustermann et al., 2011; Elsasser et al., 2012) to rescue transcriptional defects observed in DAXX-deficient cells (Michod et al., 2012). It is important to note that histone chaperones are often components of chromatin remodeling complexes, such as the nucleosome remodeling and deacetylation (NuRD) and Polycomb complexes (Lai and Wade, 2011; Margueron and Reinberg, 2011). Thus, DAXX could load H3.3 while being part of a larger chromatin-remodeling complex containing histone- and/or DNA-modifying enzymes, which could cooperate with histone loading in promoting chromatin modification and transcriptional changes.

What is the evidence for a role of H3.3 loading in transcriptional regulation? Our work and other studies discussed above suggest a potential role for H3.3 in transcription and/or regulation of the transcriptional state (Ng and Gurdon, 2008; Jullien et al., 2012; Michod et al., 2012). Furthermore, H3.3 downregulation in B cells results in transcriptional repression at the *Igh* locus (Aida et al., 2013). In contrast, loss of *H3f3b* does not dramatically alter the transcriptome of mouse embryo fibroblasts (Bush et al., 2013) and HIRA deficiency in ES cells has limited impact on transcription (Goldberg et al., 2010). It is plausible that the impact of H3.3 loading on transcription could depend on the cell type, developmental stage, and environmental cues (e.g., neuronal activation, B cell differentiation stimuli, etc). In this respect, the concept of H3.3 deposition contributing to transcriptional memory at selected loci during development is particularly intriguing. Overall, there is accumulating evidence that H3.3 might regulate transcription. A key question is how. Most active genes are associated with variant nucleosomes containing H3.3 and the histone 2A variant H2A.z, which promotes nucleosome instability (Jin et al., 2009). These properties of H3.3 may explain its enrichment at bivalent genes in flies (Henikoff, 2008) and, along with H2A.z, in mammalian cells (Creyghton et al., 2008; Goldberg et al., 2010). Many genes involved in brain development and postnatal neurogenesis are characterized by bivalent chromatin (Valk-Lingbeek et al., 2004; Marino, 2005; Lim et al., 2009; Sawarkar and Paro, 2010; Schuettengruber et al., 2011; Dawson and Kouzarides, 2012). Bivalency is defined by the presence of both active and repressive histone marks, which keep genes in a poised state. These are the repressive mark trimethylated H3 lysine 27 (H3K27me3) and the active mark H3K4me3, which are generated via the action of Polycomb Repressive Complex 2 (PRC2) and Trithorax complexes, respectively. H3K27me3 has a dual role in amplification of PRC2-mediated K27 methylation and recruitment of the Polycomb repressive complex 1 (PRC1), which mediates ubiquitylation of H2A (another repressive mark) (Wang et al., 2004). H3K4me3 is found associated with several chromatin-remodelers, as well as H3K27 demethylases (Dawson and Kouzarides, 2012). Polycomb group (PcG) and Trithorax group (TrxG) proteins are key regulators of stem cell fate in both the embryonic and postnatal brain (Simon and Kingston, 2009; Margueron and Reinberg, 2011). In particular, the PRC1 component Bmi1 and the TrxG complex component Mll1 are important regulators of neural stem cell self-renewal and neurogenesis (Valk-Lingbeek et al., 2004; Marino, 2005; Lim et al., 2009). Notably, H3.3 is enriched in the H3K4me3 mark (Henikoff, 2008). Furthermore, H2A.z has been proposed to regulate targeting of both PcG and TrxG complexes to chromatin (Hu et al., 2012). Overall, these studies suggest a potential functional involvement of H3.3 and H2A.z loading in regulation of bivalency. In particular, it is conceivable that H3.3 deposition could affect bivalent domains at IEGs in neurons, as potential mechanism for DAXX-mediated transcriptional changes (Michod et al., 2012). In general, it is of key importance to generate new genetic systems to better define the molecular function of H3.3 and its impact on fundamental biological processes. In this respect, Jeffrey Mann’s group has been involved in generation of new models based on conditional allelic replacement, which bear great promise for advancing our understanding of H3.3 function *in vivo* (Tang et al., 2013).

## H3.3 Loading and Disease Pathogenesis

An even greater interest in H3.3 and its chaperones has arisen from the discovery that H3.3 itself, DAXX and ATRX are mutated in human cancer. In this respect, driver heterozygous mutations in the *H3F3A* gene are found in pediatric glioblastoma multiforme (GBM) (Schwartzentruber et al., 2012; Sturm et al., 2012; Wu et al., 2012) (*H3F3B* is expressed at much lower levels in neural cells; our unpublished observation). H3.3 is mutated at K27 (K27M) and G34 (G34R or V), with the former found in brainstem tumors of young children (Schwartzentruber et al., 2012; Sturm et al., 2012; Wu et al., 2012) and the latter in the cerebral hemispheres of older children and adolescents (Schwartzentruber et al., 2012; Sturm et al., 2012; Wu et al., 2012). ATRX is mutated in pediatric (Schwartzentruber et al., 2012) and adult GBM (Heaphy et al., 2011), and DAXX in pediatric GBM, albeit very infrequently (Schwartzentruber et al., 2012). ATRX is also found mutated in neuroblastoma, while both DAXX and ATRX are mutated in neuroendocrine tumors of the pancreas (Elsasser et al., 2011; Heaphy et al., 2011; Jiao et al., 2011; Molenaar et al., 2012). Most DAXX and ATRX mutations are mutually exclusive and result in loss of expression (Elsasser et al., 2011; Heaphy et al., 2011; Jiao et al., 2011; Schwartzentruber et al., 2012; Wu et al., 2012), apart from a missense mutation found in acute myeloid leukemia (AML) (Ding et al., 2012). It is important to note that pediatric GBM also display mutations of ATRX in the absence of H3.3 mutations, as observed in adult GBM, neuroblastoma and pancreatic tumors, suggesting that alterations in loading of WT H3.3 may *per se* lead to cancer. H3.3/ATRX- and ATRX-only-mutated GBM tumors often carry p53 mutations, suggesting that loss of p53 tumor suppressive function cooperates with H3.3 and/or ATRX mutations for tumorigenesis. Finally, in pancreatic tumors carrying DAXX mutations, it is conceivable that H3.3 loading could be mediated by other chaperones, thus leading to alterations in its genome-wide distribution, with potential consequences for tumorigenesis. At present, it is unclear what are the expression levels of the H3.3 chaperones HIRA and DEK in pancreatic tumors and other cancers displaying alterations in H3.3 and DAXX/ATRX loading machinery.

The key question is how alterations of H3.3 function can drive/contribute to neoplastic transformation. Analysis of gene expression changes in GBM neoplasms carrying H3.3 mutations showed that H3.3 K27M and G34R/V tumors display distinct transcriptional changes. In this respect, H3.3K27M GBM tumors display deregulation of some PcG targets (Schwartzentruber et al., 2012). Furthermore, mutations of the TrxG component multiple endocrine neoplasia type 1 (MEN1) are found in neuroendocrine pancreatic tumors and is mutually exclusive with DAXX and ATRX mutations, suggesting similar functional roles (Jiao et al., 2011). Together, these data indicate that alteration of bivalent gene expression may represent one of the mechanisms underlying the transforming role of the H3.3 K27M mutation. It is important to note that deregulation of the machinery controlling the H3K27me3 epigenetic mark has been implicated in pathogenesis of another pediatric brain tumor, medulloblastoma, and adult GBM. In most cases, this leads to increased H3K27me3 (Bruggeman et al., 2007; van Haaften et al., 2009; Jones et al., 2012; Lu et al., 2012; Robinson et al., 2012), via deregulated expression/mutations of H3K27me3 methylases/demethylases, inactivation of chromatin-remodeling factors or metabolic enzymes (Bruggeman et al., 2007; van Haaften et al., 2009; Jones et al., 2012; Lu et al., 2012; Robinson et al., 2012). Increased H3K27me3 in medulloblastoma and adult GBM is expected to lead to increased repression. However, it was unclear what would be the consequences of pediatric GBM mutation of H3.3 at K27 on H3K27me3 and transcription. A very recent study from David Allis group has provided important clues (Lewis et al., 2013). In particular, this work shows that the presence of H3.3K27M negatively affects PRC2-mediated amplification of K27 trimethylation in *cis* and *trans*. This occurs via inhibition of the enzymatic activity of the PRC2 methyltransferase EZH2. Interestingly, introduction of K-to-M mutations at other known methylated H3 residues (H3K9 and H3K36) has similar negative effects on enzymatic activity of the dedicated methyltransferases. Together, these data suggest that H3.3K27M acts at least in part as a gain-of-function mutant. Notably, the gain-of-function effect of the K-to-M mutation is not restricted to H3.3, as also canonical H3 is found mutated in GBM and H3K27M displays similar EZH2 inhibitory activity. What would be the effect of H3.3K27M loading on chromatin? Some clues came from another recent study, which showed that H3.3K27M is associated with loss of H3K27me3 at many loci (Chan et al., 2013), as expected based on David Allis work. However, several genomic regions gained this mark along with H3K4me3, leading to repression of genes involved in cancer development, such as the tumour suppressor *p16INK4a* (Chan et al., 2013). Thus, the consequences of this mutation on chromatin structure/modifications are more complex than previously thought.

How would G34R/V mutations function? Notably, H3.3 G34 mutations almost invariably coexist with ATRX mutations (Henikoff, 2008; Schwartzentruber et al., 2012) and are associated with the alternative lengthening of telomeres (ALT) mechanism (Heaphy et al., 2011; Bower et al., 2012; Liu et al., 2012; Lovejoy et al., 2012), a recombinogenic mechanism for telomere elongation. ALT cells contain a modified PML-NB called ALT-associated PML-NB (APB), which we and others have implicated in telomeric damage response and potentially telomere recombination (Stagno D’Alcontres et al., 2007; Lovejoy et al., 2012). Thus, it is conceivable that H3.3 G34 mutations could lead to ATRX loss and alteration of telomere maintenance mechanisms, thus in turn contributing to transformation and GBM development. Recent work from Chris Jones laboratory shows that H3.3G34 mutations alter transcription and enrichment of the H3K36me3 active mark at a number of developmentally regulated genes linked to forebrain development and stem cell self-renewal (Bjerke et al., 2013). Remarkably, these mutations lead to increased expression of the MYCN proto-oncogene (Bjerke et al., 2013), suggesting a potential link between histone variant loading and MYCN-mediated transformation.

Are H3.3 mutations transforming *per se*? It is important to note that H3.3K27M is unable to promote glioma even in a p53 null background (Lewis et al., 2013), suggesting that either other genetic events are needed or, more likely, the cell targeted in this model is not the correct one. In this respect, it is possible that the type of progenitor and/or the developmental stage are crucial for transformation by H3.3 mutant proteins. Definite answers to these outstanding questions will be achieved only upon development of more sophisticated genetic models, which are currently in the pipeline in many laboratories in the field.

Alterations of the H3.3 chaperone complex might extend to non-neoplastic conditions, such as the ATR-X syndrome, which is driven by ATRX mutations (Gibbons and Higgs, 2000). Furthermore, ATRX interacts with MeCP2 and cohesin, mutated in the Rett and Cornelia de Lange (CdLS) syndromes, respectively (Kernohan et al., 2010). It is presently unknown whether alterations of H3.3 loading may participate in the pathogenesis of these conditions.

## Role of PML and PML-RARα in Regulation of H3.3 Loading?

**Figure 2:**
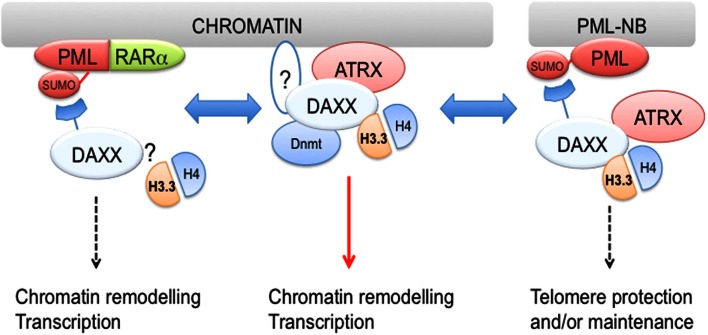
**DAXX associates with both PML and PML-RARα?**. DAXX and H3.3/H4 dimers are found at PML-NBs, suggesting that PML may regulate H3.3 loading. Furthermore, DAXX association with PML-RARα is required for transformation *in vitro*. Although it is presently unknown whether H3.3 also associates with PML-RARα, it is conceivable that PML-RARα via interaction with DAXX could modulate H3.3 loading. While DAXX is recruited to PML-NBs via a SUMO-interacting motif (SIM)-dependent mechanism, it is still unclear whether a similar mechanism is implicated in its targeting to chromatin (see question mark in middle panel). DAXX also interacts with DNA methyltransferase 1 (Dnmt1) and targets its activity to chromatin, suggesting that DAXX coordinates multiple epigenetic modifications.

As mentioned earlier, DAXX localizes to PML-NB via a SUMO-dependent mechanism involving its SIM (Figure 2). Thus, it is conceivable that PML could regulate DAXX function by altering its subcellular localization. In this respect, PML was reported to negatively regulate DAXX repressive function through recruitment to PML-NBs (Li et al., 2000). However, PML role in regulation of H3.3 deposition is still unclear. Clues have come from a recent study reporting H3.3/H4 dimers localization to PML-NBs (Figure 2) (Delbarre et al., 2012). The authors of this study report that exogenously expressed H3.3 along with H4 and DAXX localizes to PML-NBs in G1-enriched mesenchymal stem cells, thus potentially regulating the nucleoplasmic pool of H3.3. Although exogenous H3.1 and H3.2 failed to localize to PML-NBs, it is still possible that H3.3 could be targeted to PML-NBs only when expressed at supraphysiological levels. One obvious question is whether PML regulates incorporation of endogenous H3.3 into chromatin. In this respect, it could hypothesized that PML-mediated localization of H3.3 and its chaperones to PML-NBs inhibits H3.3 loading into chromatin (Delbarre et al., 2012). Another recent report implicated PML and PML-NBs in regulation of ATRX and H3.3 association at telomeres during S-phase in ES cells (Chang et al., 2013). As a result, PML downregulation caused telomere dysfunction and altered telomeric enrichment of selected epigenetic marks. Although it cannot be excluded that PML-NBs are sites for localization of extrachromosomal telomeric DNA, this work suggests that PML is either directly or indirectly involved in regulation of telomere replication/maintenance potentially via an H3.3/ATRX-dependent mechanism. A question arising from this study is whether PML role in regulating H3.3 association with telomeric DNA is maintained also in ALT cells, which are often ATRX-negative, in particular in G34R/V, ATRX-deficient GBM cells. Finally, PML could play a more indirect role in regulation of H3.3 loading via modulation of DAXX PTMs, in particular its phosphorylation at S669 (Michod et al., 2012). In this respect, while the S669 phosphatase CaN is mainly cytosolic, the S669 kinase HIPK2 has been shown to localize to PML-NBs (Krieghoff-Henning and Hofmann, 2008), suggesting that localization of HIPK2 and DAXX to PML-NBs could affect its phosphorylation and as a result its chaperone activity.

Promyelocytic leukemia-mediated regulation of DAXX function could be particular relevant in the central nervous system (CNS), given the roles played by the two proteins in this context. In this respect, our previous work has shown that PML is expressed in neural progenitor/stem cells (NPCs) in the developing neocortex as well as in postnatal neurogenic niches [(Regad et al., 2009; Salomoni and Betts-Henderson, 2011) and our unpublished data]. As a result, PML loss leads to alterations of corticogenesis and smaller brains (Regad et al., 2009), as well as aberrant postnatal neurogenesis (our unpublished data). While PML and DAXX are expressed in the germinal area of developing neocortex and in NPCs within adult neurogenic niches, PML expression is downregulated in postmitotic neuroblasts and neurons (Regad et al., 2009; Michod et al., 2012) (and our unpublished data). It is therefore possible that PML could regulate DAXX chaperone function in NPCs, thus potentially affecting epigenetic changes driven by H3.3 loading. In turn, this could have implications for cell fate regulation and neurogenesis. In contrast, PML-mediated control of DAXX function would be absent in differentiated neurons.

What about the oncogenic form of PML, PML-RARα? SUMOylation within the PML moiety of PML-RARα is required for transformation (Zhu et al., 2005) and is responsible for recruitment of DAXX via its C-terminal SIM (Figure 2; see also accompanying review articles by Hugues de The in this issue of Frontiers). Mutation of the critical SUMOylation site within PML-RARα (K160R) releases DAXX and results in defective differentiation block (Zhu et al., 2005). In contrast, fusion of K160R PML-RARα with DAXX restores its transforming capacity (Zhu et al., 2005). A subsequent study from de The’s laboratory reported that a DAXX-RARα chimera carrying a multimerization domain can repress RA-dependent transcription, inhibit differentiation, and promote transformation. In contrast, a multimerization-prone RARα mutant, despite inhibiting RA-dependent transcription and differentiation, was unable to transform hematopoietic progenitors (Zhou et al., 2006), suggesting that molecular determinants of the differentiation block and transformation may not be identical. However, a separate study by Eric So’s group showed that fusion of the FKBP oligodimerisation sequence with RARα can promote transformation (Kwok et al., 2006). The presence of a SUMOylation site (Rodriguez et al., 2001) in FKBP (which would in principle still recruit DAXX) and non-identical experimental settings may explain the different results. Overall, these findings suggest that SUMO-dependent PML-RARα association with DAXX contributes to block of differentiation and transformation.

PML-RARα has been reported to associate with epigenetic regulators and mediate epigenetic changes: (i) PML-RARα multimerization properties lead to increased density of corepressors and chromatin remodeling factors at retinoic acid (RA) target genes (Lin and Evans, 2000; Minucci et al., 2000; de The and Chen, 2010); (ii) PML-RARα interacts with DNA methyltransferases, thus leading to DNA methylation of a number of RA target genes (Di Croce et al., 2002). Interaction with the H3.3 chaperone DAXX could provide PML-RARα with additional weaponry to promote epigenetic changes. One could argue that H3.3 is mainly associated with active genes, not with repression. In this respect, it is important to note that there is little *in vivo* evidence that repression of RA target genes is sufficient to initiate APL (de The and Chen, 2010), and it is now recognized that PML-RARα also possess gain-of-function properties through its ability to bind target sequences that are not recognized by the normal RARα-RXRα heterodimers (de The and Chen, 2010). Among these sites, there are many genes controlling stem cell self-renewal and myeloid differentiation (Purton et al., 2006; Viale et al., 2009; de The and Chen, 2010). Finally, mouse APL leukemias express high levels of the IEG c-Fos (Yuan et al., 2007), which we have shown to be regulated by DAXX in neural cells (Michod et al., 2012). Overall, the existing literature suggests that PML-RARα promotes transformation through a combination of dominant-negative and gain-of-function activities. Interaction with DAXX could contribute to the latter.

Considering H3.3 enrichment at bivalent genes and the alteration of PcG/TrxG activities in hematopoietic tumors (Mills, 2010; Muntean and Hess, 2012), it could be hypothesized that DAXX-mediated H3.3 loading could affect bivalent gene expression in APL cells. In this respect, there is a functional crosstalk between RA-dependent transcription and the PcG machinery, as many homeobox genes contain RA responsive elements (RARE) (Mainguy et al., 2003; Ringrose and Paro, 2004) and RA targets are also PcG targets (e.g., CYP26a1 and RARβ). Notably, PML-RARα-regulated loci display increased H3K4me3 (Hoemme et al., 2008), thus suggesting that PML-RARα may regulate this epigenetic mark. As H3.3 is enriched in H3K4me3, it is conceivable that PML-RARα could direct DAXX-mediated H3.3 deposition at a number of its gene targets. As H3K4me3 is lost from a number of bivalent loci during differentiation of human hematopoietic stem cells (Cui et al., 2009), it is possible that PML-RARα could re-establish/maintain H3K4me3 at these loci through modification of TrxG complex activity and/or loading of H3.3. PML-RARα is also found in complex with PRC2 components (Villa et al., 2007; Martens et al., 2010), suggesting it could affect H3K27 trimethylation. However, subsequent genome-wide analysis showed that RA treatment fails to significantly affect H3K27me3 (Martens et al., 2010). More work is needed to define PML-RARα role in regulation of bivalent chromatin and the contribution of H3.3 loading to its epigenetic activity. It is important to note that a DAXX missense mutation of unknown functional consequences has been identified in AML (Ding et al., 2012), suggesting that alterations of H3.3 loading may occur in non-APL hematopoietic neoplasms.

Finally, both PML and PML-RARα have been linked to another H3.3 chaperone, HIRA (Ye et al., 2007; Delbarre et al., 2012). In this respect, localization of HIRA to PML-NBs is required for formation of senescence-associated heterochromatic foci (SAHF). PML-RARα, which disrupts PML-NBs, inhibits SAHF generation. Although it is presently unknown whether H3.3 is loaded as SAHF, these data suggest that PML and its oncogenic version may regulate H3.3 loading by acting on multiple chaperones.

## Conclusion

**Figure 3:**
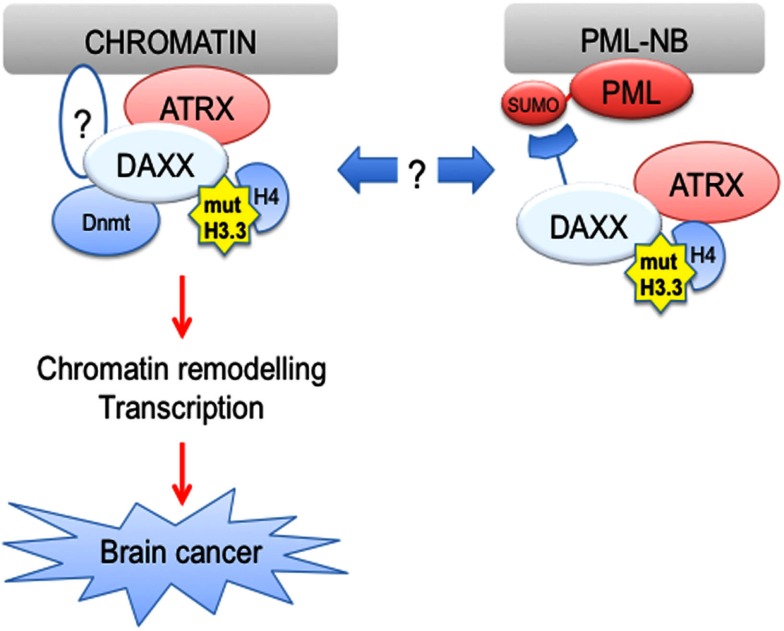
**H3.3 is mutated in human cancer**. Driver H3.3 mutations are found in pediatric glioblastoma (pGBM), suggesting that alterations of H3.3 function may lead to brain cancer. It is possible that PML via its ability to recruit DAXX to PML-NBs could regulate loading of mutant H3.3 proteins, thus potentially affecting brain tumorigenesis.

The discovery of DAXX chaperone function provides the fascinating possibility that PML and its oncogenic form PML-RARα could promote epigenetic changes in part via regulation of H3.3 loading. In this respect, PML-RARα could utilize DAXX chaperone activity to modify the epigenetic and transcriptional status of its target genes, as part of its gain-of-function activities in transformation of hematopoietic progenitors. In contrast, PML could play a more indirect role in regulation of loading of wild-type H3.3 as well as its GBM-associated mutants by controlling the availability of soluble H3.3/H4 dimers and/or DAXX PTMs, with potential implications for cell fate regulation and transformation (Figure 3). In this respect, pharmacological degradation of PML via ATO treatment could represent a strategy to affect H3.3 loading in cancer cells. More broadly, an increased understanding of H3.3 loading, its function and regulatory pathways has the potential to lead to a paradigm shift in the field of cancer epigenetics.

## Conflict of Interest Statement

The authors declare that the research was conducted in the absence of any commercial or financial relationships that could be construed as a potential conflict of interest.

## Footnotes

^1^http://www.frontiersin.org/Molecular_and_Cellular_Oncology/researchtopics/The_PML_protein_a_tumor_suppre/713
